# FIP1L1-PDGFRA Clonal Hypereosinophilic Syndrome With Eosinophilic Myocarditis and Intracardiac Thrombus

**DOI:** 10.7759/cureus.43138

**Published:** 2023-08-08

**Authors:** Margaret Locke, Rachel M Suen, Alex K Williamson, Maria J Nieto

**Affiliations:** 1 Internal Medicine, Zucker School of Medicine, Hempstead, USA; 2 Internal Medicine, Mayo Clinic, Rochester, USA; 3 Pathology, Zucker School of Medicine, Hempstead, USA; 4 Hematology, Zucker School of Medicine, Hempstead, USA

**Keywords:** hypereosinophilic syndrome, eosinophilic leukemia, intracardiac thrombus, clonal hypereosinophilic syndrome, eosinophilic myocarditis

## Abstract

A 45-year-old man from El Salvador with no past medical history presented with cough and chest pain. Investigations revealed 60% peripheral eosinophilia (absolute count 12.3 K/uL). Cardiac imaging was consistent with myocarditis with intracardiac thrombus formation. Endomyocardial biopsy confirmed eosinophilic infiltration of the myocardium, and bone marrow biopsy showed hypercellular marrow with 28% eosinophils. Cytogenetics/fluorescence in situ hybridization (*FISH*) confirmed positive FIP1L1-PDGFRA rearrangement. The patient was treated for FIP1L1-PDGFRA clonal hypereosinophilic syndrome with associated eosinophilic myocarditis and intracardiac thrombus. The treatment regimen consisted of a steroid taper, imatinib, and anticoagulation. Treatment was followed by normalization of the eosinophil count. At two-year follow-up, the patient was without recurrence of eosinophilia on maintenance imatinib and indefinite anticoagulation with warfarin.

## Introduction

Hypereosinophilic syndromes comprise a rare and heterogenous group of disorders. Linked by the criteria for a blood eosinophil count of at least 1500 cells/m^2^ and evidence of eosinophilic organ involvement, etiologies range from infectious (parasitic), allergic, rheumatologic, to neoplastic [1,2]. Clonal hypereosinophilic syndromes have been documented to be associated with a chromosome 4q12 deletion, resulting in a constitutively active tyrosine kinase, as is the case in a FIP1L1-PDGFRA rearrangement [3]. Prevalence of the FIP1L1-PDGFRA+ clonal hypereosinophilic syndrome has been estimated at between 0.31 and 6.3 cases per 1,000,000 people [4]. The most common organs involved are the spleen followed by the lungs. Cardiac involvement is uncommon, with few case reports of myocarditis in the literature. Given the significant morbidity and mortality associated with eosinophilic myocarditis, prompt identification and treatment initiation are crucial to prevent progression of heart failure and thrombotic events [5]. Here, we discuss a rare case of a patient with FIP1L1-PDGFRA clonal hypereosinophilic syndrome with eosinophilic myocarditis in the thrombotic phase successfully treated with imatinib.

## Case presentation

**Figure 1:**
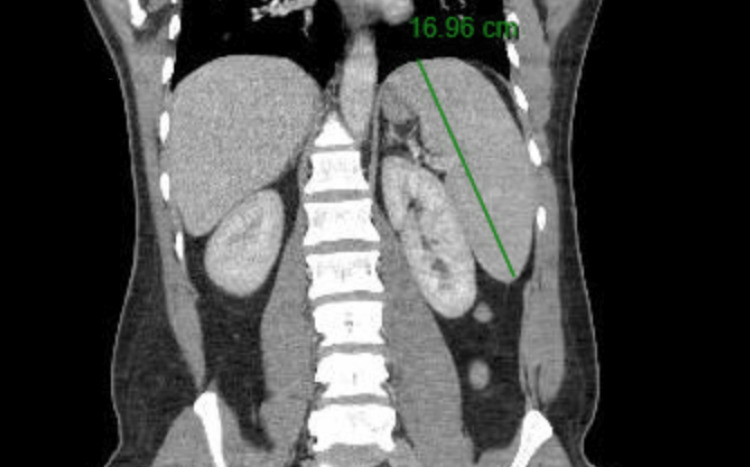
CT abdomen/pelvis with contrast showing splenomegaly (green line).

A 45-year-old man from El Salvador with no past medical history presented with two months of dry cough and two days of chest pain. Vitals on admission were notable for sinus tachycardia to 140 beats per minute. Initial investigations on admission found elevated high-sensitivity troponin of 2123 (normal 0-14), pro B-type natriuretic peptide (BNP) of 14,000, and marked peripheral eosinophilia of 12.3 K/uL (60.0%). Hemoglobin was 14.4 K/uL, and platelet count was 207 K/uL. CT imaging of the head, chest, and abdomen/pelvis was negative for pulmonary embolism but showed splenomegaly to 17 cm in the maximum dimension and scattered sub-centimeter lymph nodes (Figure 1).

**Figure 2:**
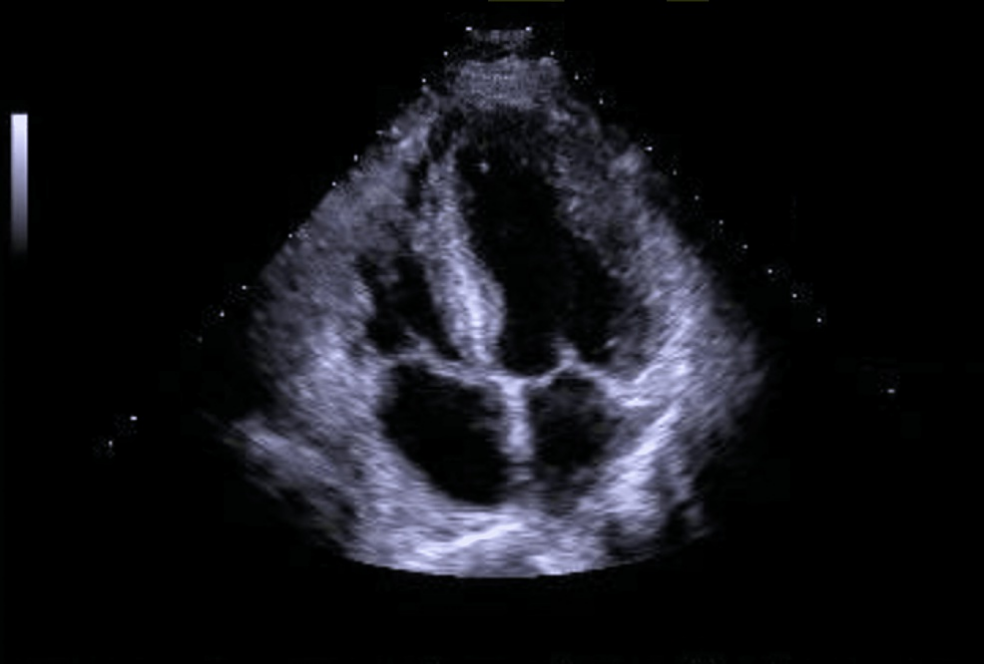
Four-chamber transthoracic echocardiogram showing mild left ventricular hypertrophy.

Transthoracic echocardiogram revealed left ventricular ejection fraction (LVEF) of 53% with mild concentric left ventricular (LV) hypertrophy and -7.6% global longitudinal strain (GLS) (Figure 2).

The patient was admitted to the cardiac intensive care unit for close monitoring of presumed eosinophilic myocarditis.

Investigations

**Figure 3:**
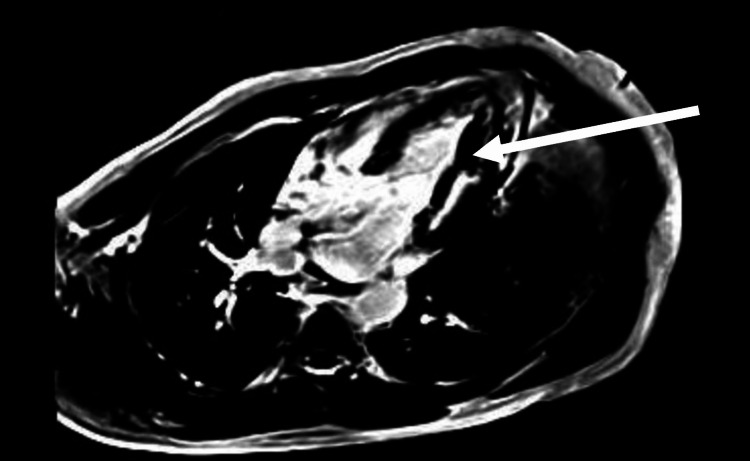
Phase-sensitive inversion recovery (PSIR) MRI image showing a layer of thrombus overlaying the lateral left ventricle.

Cardiac MRI revealed mildly reduced LVEF 45% and diffuse late gadolinium enhancement over the lateral wall of the left ventricle with a layer of thrombus and papillary muscle involvement (Figure 3). 

**Figure 4:**
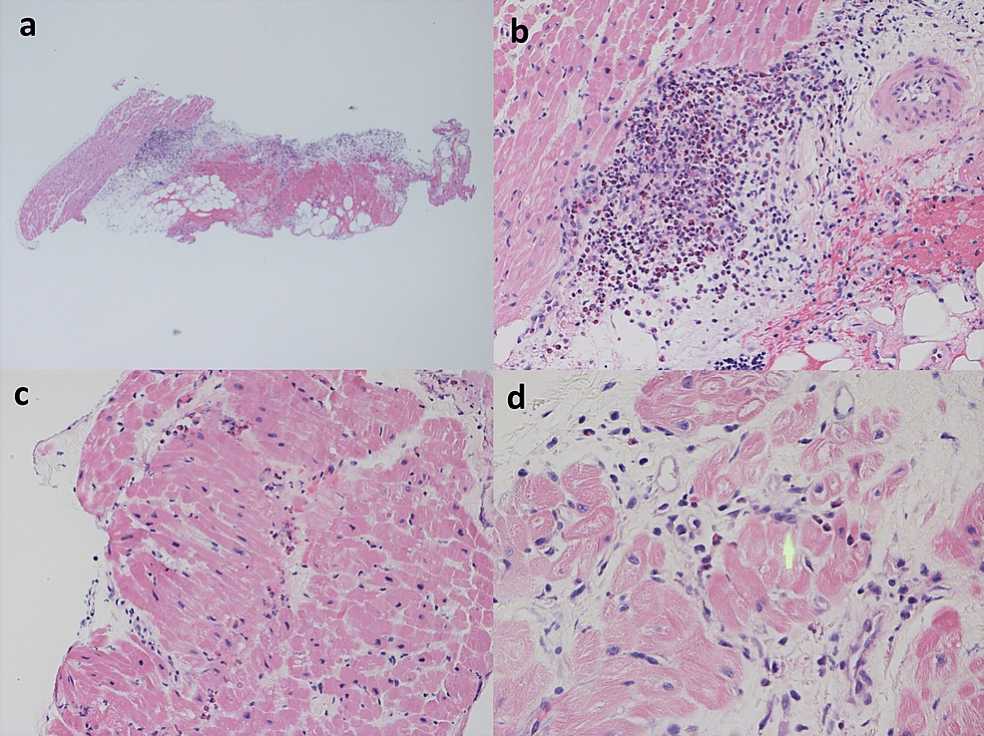
a) Full-thickness section of the right ventricle wall including the endocardium (to left), myocardium, and epicardial fat surfaced by the mesothelium and containing a hemorrhage (to right), showing a collection of leukocytes, including numerous eosinophils at the myocardial-epicardial interface and extending into the myocardium and epicardial fat. Hematoxylin and eosin (H&E), 20x. b) Higher-magnification view of image 1 showing a collection of leukocytes, including numerous eosinophils at the myocardial-epicardial interface. H&E, 200x. c) Myocardium with eosinophils throughout the interstitium. H&E, 200x. d) Myocardium with eosinophils throughout the interstitium and focally associated with a myocyte injury (at the green arrow). H&E, 400x.

Infectious workup for strongyloidiasis, schistosomiasis, trichinellosis, filariasis, and stool ova and parasites were negative, but toxocara canis antibody was positive. C3/C4 were normal, immunoglobulin E (IgE) was 186 KU/L, vitamin B12 was >2000 pg/mL, and tryptase was elevated to 19.9 ug/L. The patient was negative for HIV, anti-nuclear antibody (ANA), and anti-neutrophil cytoplasmic antibodies (ANCA). Cardiac catheterization found that coronary arteries were without stenosis. Endomyocardial biopsy was also performed, which showed numerous eosinophils, few neutrophils, and some mononuclear leukocytes extending throughout the epicardial fat and focally infiltrating the outer myocardium (Figure 4). 

Bone marrow biopsy showed a hypercellular marrow with myeloid hyperplasia, eosinophilia (28%), myeloid and erythroid maturation, mild increase in megakaryocytes and mast cells, and increased iron stores. Flow cytometry showed trilineage hematopoesis and significant eosinophilia. There was no blast population, and myeloid antigen pattern and granularity were normal. Cytogenetic/fluorescence in situ hybridization (FISH) testing showed that FIP1L1-PDGFRA 4q12 rearrangement was positive. BCR/ABL, c-kit, and JAK2 were negative.

Treatment

The patient was diagnosed with clonal hypereosinophilic syndrome with eosinophilic myocarditis, and he was started on prednisone. Given the presence of the FIP1L1-PDGFRA 4q12 rearrangement, the patient was also started on imatinib while inpatient. The patient completed a five-day course of albendazole due to positive toxocara serologies. Anticoagulation was also started for the intracardiac thrombus, first with a heparin drip then transitioned to warfarin. Steroids were tapered over many months as an outpatient, and the patient also received trimethoprim-sulfamethoxazole for *Pneumocystis *prophylaxis while on steroids.

Outcome

Initiation of the therapies achieved a sustained complete response. Treatment was followed by normalization of the eosinophil count a few days later (eosinophil count peaked at 18.8 K/uL). The patient was tapered off of prednisone as an outpatient but continued on maintenance imatinib and indefinite anticoagulation with warfarin. At the two-year follow-up appointment after the initial presentation, the patient’s complete blood counts remained stable without recurrence of peripheral eosinophilia. Follow-up transthoracic echocardiogram at this time showed resolution of the previously seen LV thrombus; however, hypokinesis of the inferior and inferolateral walls persisted, LVEF was 50%, and GLS recovered to -16.1%.

## Discussion

The tyrosine kinase inhibitor, imatinib, has a revolutionized treatment for FIP1L1-PDGFRA+ clonal hypereosinophilic syndrome. Studies following the long-term outcomes of affected patients have found complete hematological response in >90% of cases [6]. Although there are no formal guidelines for the duration and discontinuation of imatinib therapy, a small number of studies have reported the median time to relapse, ranging from 17 to 30 months after discontinuation of imatinib [6,7]. Given that imatinib was well tolerated by the patient of this case, imatinib was planned to be continued indefinitely.

Cardiac imaging is a cornerstone in the identification and monitoring of eosinophilic myocarditis. The natural progression of eosinophilic myocarditis is well documented to occur in three phases: necrotic, thrombotic, and fibrotic [5]. At early stages, as was the case in this patient in the thrombotic phase, changes related to subendocardial eosinophilic infiltration are more subtle and may not be readily detected on transthoracic echocardiogram. Thus, cardiac MRI is a key tool in diagnosis. In this case, a final diagnosis of eosinophilic myocarditis was well supported and confirmed by both MRI and endomyocardial biopsy results. However, there are some cases where diagnosis may be made based off significant peripheral eosinophilia and characteristic MRI imaging alone after a discussion of risks and benefits of obtaining an endomyocardial biopsy [8]. In addition, the transthoracic echocardiogram obtained at the two-year follow-up showed segmental hypokinesis, but thrombus and strain resolution were overall consistent with the natural progression to the fibrotic phase of eosinophilic myocarditis.

Currently, there are no guidelines regarding the optimal duration of anticoagulation therapy in thrombosis associated with clonal hypereosinophilic syndromes. Several mechanisms drive the prothrombotic state associated with hypereosinophilia. Eosinophils release cytokines that increase the production of thrombin and release of platelet-activating factor. In addition, the endomyocardial fibrosis that accompanies eosinophilic myocarditis enhances the risk of developing intracardiac mural thrombus [9]. Routine anticoagulation is not recommended unless the patient develops intracardiac thrombus and/or thromboembolic event. In cases of LV thrombus not associated with an underlying myeloprofilerative neoplasm, discontinuation of anticoagulation is considered after three months. In this case, however, lifelong anticoagulation was planned as the presence of the FIP1L1-PDGFRA rearrangement poses a high risk for disease recurrence and progression. Early data have suggested that the risk for thrombotic events remains elevated even after normalization of the eosinophil count, suggesting that the degree of disease activity is an important risk factor [10]. Finally, although vitamin K antagonists are most commonly used for intracardiac thrombus, reports have suggested that direct oral anticoagulants may be safely used as an alternative [11].

Etiologies driving hypereosinophilic syndromes are broad, and this patient underwent thorough an infectious workup. Interestingly, *Toxocara canis* antibodies returned positive. Although toxocariasis can cause eosinophilia, marked elevation to levels found in this case would be unusual. In addition, the toxocara immunoglobulin G (IgG) may more likely be a marker of a past or resolved infection, as it can be persistently positive for years after resolution. Regardless, the patient in this case was treated with albendazole empirically given the severity of disease.

## Conclusions

FIP1L1-PDGFRA+ clonal hypereosinophilic syndrome is a rare disorder that can present with organ infiltration and, uncommonly, eosinophilic myocarditis. As demonstrated in this case, prompt identification with advanced cardiac imaging, pathology, and cytogenetics are of utmost importance because affected patients are good candidates for imatinib therapy. Additional research is needed to evaluate the optimal duration of anticoagulation therapy for patients with complete hematological remission in response to imatinib.
